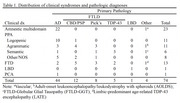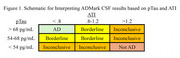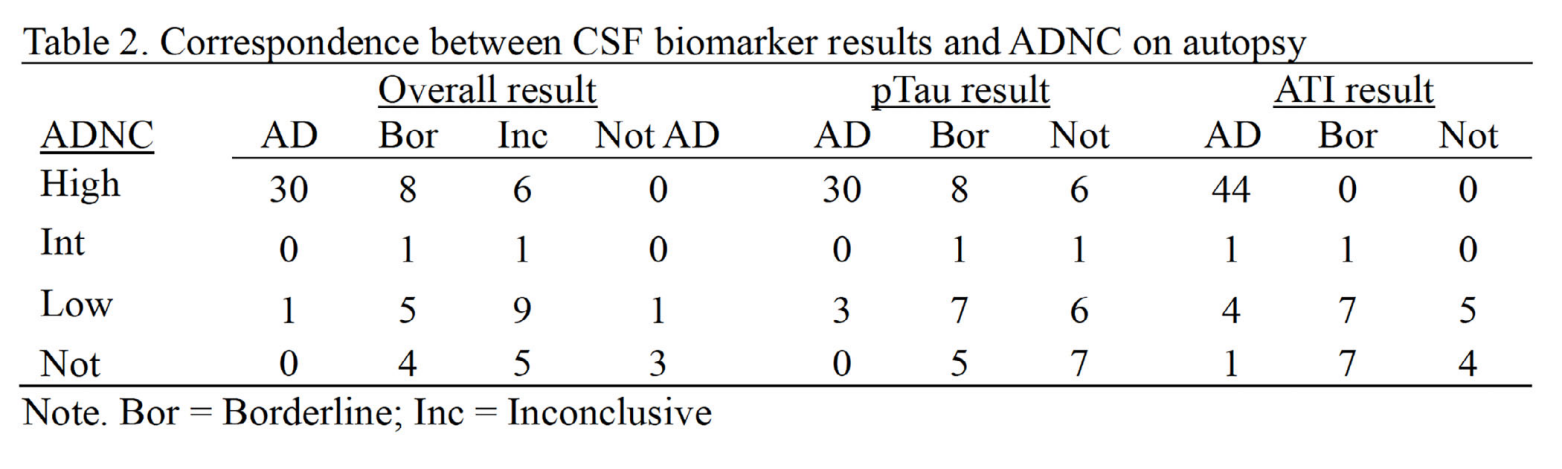# Correspondence between borderline and inconclusive pre‐mortem CSF Alzheimer's disease biomarkers and post‐mortem Alzheimer's disease neuropathologic change

**DOI:** 10.1002/alz70856_105984

**Published:** 2026-01-09

**Authors:** Molly A Mather, Allison Lapins, Nina Reiser, Deborah Zemlock, Tamar Gefen, Sandra Weintraub, Changiz Geula, David Gate, Marsel Mesulam, Robert J. Vassar, Joshua Gabriel Cahan

**Affiliations:** ^1^ Northwestern University Feinberg School of Medicine, Chicago, IL, USA; ^2^ Mesulam Center for Cognitive Neurology & Alzheimer's Disease, Chicago, IL, USA; ^3^ Abrams Research Center on Neurogenomics, Chicago, IL, USA

## Abstract

**Background:**

Positive CSF biomarkers have been shown to be a reliable predictor of post‐mortem Alzheimer's disease neuropathologic change (ADNC) and are often incorporated into clinical diagnosis of neurodegenerative disease. However, less is known about the predictive value of borderline or inconclusive results, which are not uncommon in clinical practice. The current project determined correspondence between CSF AD biomarkers and post‐mortem ADNC in a clinical research sample enriched for atypical dementia syndromes, focusing on borderline and inconclusive results.

**Method:**

All participants in the Northwestern University Alzheimer's Disease Research Center (NUADRC) with available CSF AD biomarker results who donated their brain at the time of death were included (*N* = 74, Mean age at CSF = 64.99 [SD = 7.34], Mean years from CSF to death = 5.6 [range 1‐14 years], 43% female, 95% non‐Hispanic white; see Table 1 for breakdown of clinical syndromes and pathological diagnoses). CSF results were categorized as AD, Borderline, Inconclusive, or Not AD based on ADMark thresholds for amyloid‐b_42_/total tau index (ATI) and phosphorylated tau (pTau; Figure 1).

**Result:**

Across clinical phenotypes, results consistent with AD on CSF were highly specific for High ADNC on autopsy (97%) but only moderately sensitive (68%); positive predictive value was also high (97%). Within Borderline and Inconclusive cases, abnormal ATI reliably predicted High ADNC on autopsy (sensitivity = 100%, specificity = 80%), regardless of pTau results. In this sample, there were no cases with pTau in the AD range but normal ATI that had High ADNC on autopsy. Abnormal CSF ATI and/or pTau were seen in some cases of FTLD‐tau, FTLD‐TDP, and other primary neuropathologic diagnoses.

**Conclusion:**

Consistent with prior findings, CSF results categorized as consistent with AD were highly specific and predictive of primary ADNC pathology on post‐mortem analysis, regardless of clinical presentation. In the setting of Borderline or inconclusive results, ATI in the AD range reliably predicted high ADNC, regardless of pTau range. These findings help clarify the prognostic utility of ATI and pTau values in Borderline and Inconclusive CSF results, which can help inform differential diagnosis between AD and other neurodegenerative etiologies.